# Low cost and long-focal-depth metallic axicon for terahertz frequencies based on parallel-plate-waveguides

**DOI:** 10.1038/s41598-021-82503-x

**Published:** 2021-02-04

**Authors:** A. I. Hernandez-Serrano, Emma Pickwell-MacPherson

**Affiliations:** Department of Physics, University of Warwick, Gibbet Hill Road, Coventry, CV4 7AL UK

**Keywords:** Terahertz optics, Optoelectronic devices and components

## Abstract

In this work we demonstrate a triangular surface lens (axicon) operating at frequencies between 350 and 450 GHz using parallel-plate-waveguide technology. The proposed axicon offers longer focal depth characteristics compared to conventional plastic lenses, surpassing common TPX lenses by one order of magnitude. Additionally, due to the triangular surface of the axicon, this device is able to focus THz radiation onto smaller areas than TPX lenses, enhancing the resolution characteristics of THz imaging systems. The frequency range of operation of the proposed axicon can be easily tuned by changing the space between plates, making this approach a very attractive candidate for low-cost, robust and easy to assemble solutions for the next generation of active THz devices.

## Introduction

Terahertz light (100 GHz up to 10 THz) has a wide range of potential applications, including but not limited to medical inspections^1,2^, industrial quality control^3,4^ and wireless communications^5–7^. Due to the versatility of this part of the electromagnetic spectrum, tremendous effort has been made towards the fabrication of devices capable of manipulating this light efficiently. In recent years, 3D printing has exhibited sufficient capabilities for the generation of new and exotic geometries for THz devices^8,9^. Unfortunately, due to the resolution of convectional 3D printers (~ 100 μm), functional 3D printed devices usually operate at frequencies below 500 μm. Recently, the use of parallel-plate waveguide structures (PPWG) have been shown to be useful for the design of photonic devices for frequencies up to 1THz^10,11^. Due to the relatively long wavelength of THz frequencies (300 μm to 3 mm for 1 THz to 100 GHz, respectively), the required features of such devices (~ 1 mm) do not present a challenge for large-scale production. Furthermore, PPWG devices are commonly made out of stainless steel, presenting excellent robustness for operating in a range of environments. Lenses are one of the essential components in any optical system. Notable progress to fabricate lenses using PPWG technology has been made recently. Mendis et al. presented a multi-stack array of metallic plates with curved surfaces able to mimic the performance of a spherical lens^12^. Additionally, Hernandez et al. were able to produce a gradient-refractive-index (GRIN) lens using the same technology^13^. In both approaches, the devices had focal lengths of less than 10 mm. Nowadays, the optical components found in THz systems are fabricated from polymer (Teflon, HDPE, TPX). Unfortunately, they encounter limitations when F-numbers closer to 1 are needed^14,15^. Additional methods for lens fabrication involve the use of diffractive elements as Fresnel zone lens, kinoforms and metamaterials^14,15^. The use of binary Fresnel zone optics has a major drawback and suffers significant scattering losses at frequencies beyond the designed frequency. This significantly reduces the range of operation of binary Fresnel zone optics and can even make them single frequency devices^16,17^. Similar drawbacks are found in the use of Fresnel zone lenses^18^. The use of kinoforms, in particular, high order kinoforms (HOK) can alleviate this disadvantage, and operate in a wider frequency range^19^. However, under certain conditions clearly explained by Sypek et al.^19^ HOK presents shifts in its focal position, defocusing the beam within its frequency range of operation. Using binary optics technology, the fabrication of axicons has been proposed^20^. As well as refractive axicons, binary optics axicons have a remarkably long depth of field. Furthermore, with the increasing demand of 3D printed THz optics, 3D printed axicons have demonstrated impressively long depths of field, even reaching 270 mm^21–23^. Similar to Fresnel zone plates, these are single frequency elements. However, they present notorious scattering losses and require precise fabrication in order to achieve the desired phase information at the design frequency. The use of metasurfaces for light control has attracted the attention of several research groups due to their unique characteristics^24,25^. Frequently, the fabrication of these structures is not trivial, making them impractical for mass production. All the devices detailed above are summarized in Table S1 in the supplementary section. Aside from the PPWG lens, they have one common characteristic, they are not tunable. Normally, the frequency range of operation of the device, once fabricated, is not able to be changed. Fortunately, parallel-plate-waveguide based devices can successfully abolish this disadvantage. In this work using a triangular geometry, we demonstrate a longer focal depth axicon compared to conventional lenses. We demonstrate that at 450 GHz the focal length of the proposed axicon can reach 30 mm. Additionally, the proposed axicon presents better resolution capabilities than spherical plastic lenses (TPX). Further numerical simulations even predict focal depths surpassing 50 mm for frequencies beyond 500 GHz. In comparison, the fabrication complexity of the proposed technology is remarkably lower than for HOK or metalenses. Furthermore, the frequency range of operation of the PPWG axicon can be tuned simply by changing the spacing between plates, surpassing the fixed bandwidth operation limitation of its diffractive/polymer optics counterparts. This work presents further evidence of the importance of PPWG-based devices in the development of novel THz devices. We envisage widespread implementation of such devices in the next generation of THz photonic components for wireless communications.

## Design and fabrication

**Figure 1:**
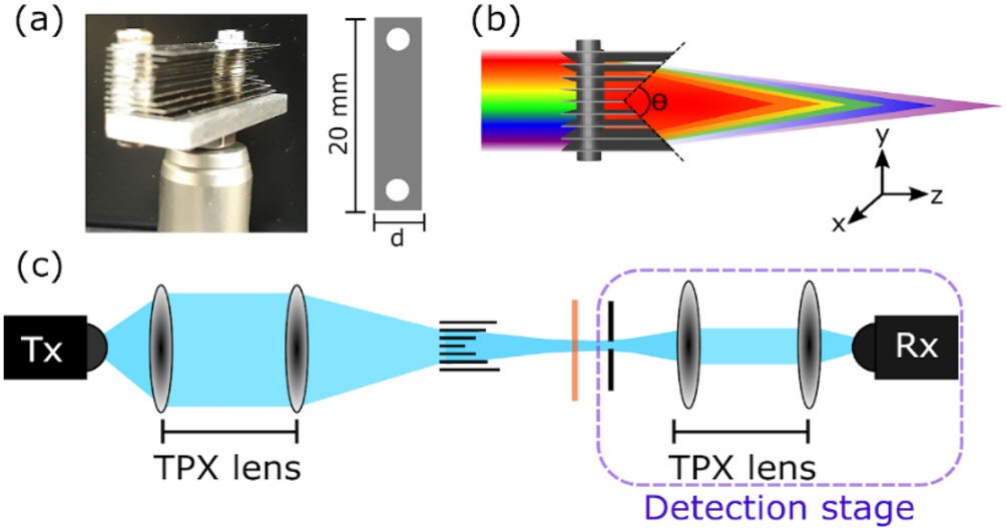
(**a**) Photo of the triangular-shaped axicon and dimensions. (**b**) False color diagram representing the variation of the focal length as function of the frequency. (**c**) Experimental setup for the investigation of focal depth and cross-section properties of the metallic axicon. The blue regions denote THz light. The sample is represented by the orange line.

The PPWG-based lens presented in this work was fabricated from stainless steel sheets with 0.1 mm thickness and 20 mm width. The length of every plate varies in a linear fashion from 5 to 10 mm in steps of 1 mm. The spacing between plates is fixed at 0.55 mm. A schematic diagram and a photo of the axicon are shown in Fig. 1a,b.

Due to the geometry of the proposed axicon, the apex angle (θ) has a value of ≈ 56°. The apex angle has a direct impact on the performance of the axicon^21–23^. In Fig. 1a the dimensions of the plates are presented: *d* takes values from 5 to 10 mm in steps of 1 mm. The thickness of each plate was kept constant at 0.1 mm. Smaller thicknesses relative to the wavelength of operation are desirable in order to reduce scattering losses, however, the choice of 0.1 mm thick plates was made to keep an acceptable balance between mechanical robustness and scattering effects. The principle of operation of the axicon resides in the fact that an effective refractive index can be attributed to each pair of plates^13^. Then a difference in phase caused by the propagation of the light inside each pair of plates produces constructive interference at the exit face focusing the energy. The phase acquired in any pair of plates is given by^13^:

1 $$\varphi =kL\sqrt{1-\frac{{c}^{2}}{4{h}^{2}{f}^{2}}},$$

in which *k* is the wavenumber in a vacuum, *L* is the length of the plate, *c* is the speed of light in a vacuum, *h* the spacing between plates and *f* the frequency. Equation (1) remains valid when only the first transverse electric mode (${TE}_{01}$) is excited. Otherwise, extra calculations have to be carried out to take into account higher order modes. In this work, the frequency range of operation guarantees single mode operation^26,27^. Due to the dependency of the phase on the frequency, as is demonstrated in Eq. (1), it is expected that different frequencies focus at different positions after the axicon, i.e. chromatic aberration limiting the frequency range of operation of the device. Due the characteristics of PPWG devices this chromatic aberration is unavoidable. This is represented by the false color diagram in Fig. 1b.

## Experimental validation

**Figure 2:**
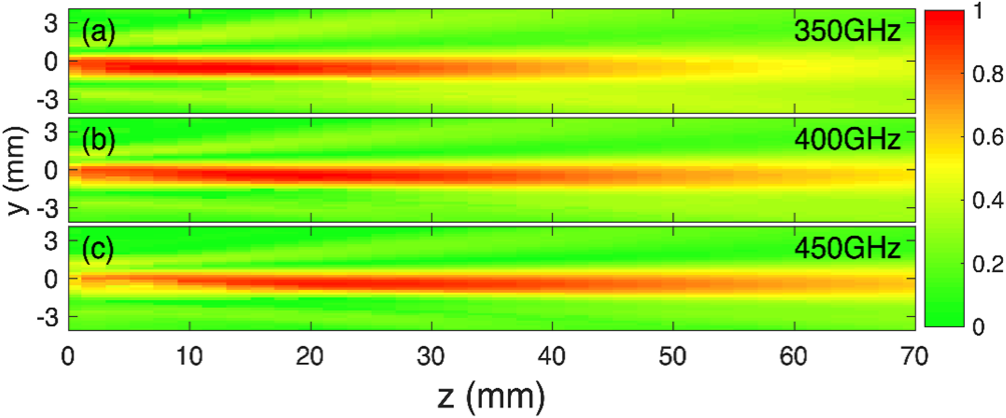
Experimental results for the longitudinal propagation of the normalized electric field at (**a**) 350 GHz, (**b**) 400 GHz and (**c**) 450 GHz. The false color image represents the amplitude of the E-field at a particular frequency. The propagation of the THz beam goes from left to right.

To examine the performance of the proposed geometry, the fibre-coupled TeraSmart THz spectrometer from Menlo systems was used. The THz system is able to deliver broadband THz pulses of 1 ps temporal duration at a dynamic range of 90 dB covering the spectral range comprised between 0.1 THz and 5 THz. The THz spectrometer was arranged in a transmission configuration similar to the one shown in^13^. A schematic diagram of the setup is shown in Fig. 1c. To examine the depth of field (DOF) of the proposed geometry, the detection stage shown in Fig. 1c has been mounted on two motorized linear stages running along the *y–z* axis, raster scanning the *y–z* plane. The results are shown in Fig. 2. The propagation of the THz beam goes from left to right.

From the experimental results it is clear that the DOF of the axicon depends on the frequency of operation, with 450 GHz being the frequency which presents the longest DOF, extending for more than 30 mm. Beyond 500 GHz, the excitation of higher order modes degrades the quality of the focused beam. These results demonstrate the capability of this structure to effectively focus the THz radiation in a similar fashion to conventional lenses. In addition, the focusing efficiency is calculated by the ratio of the power inside a 3×FWHM region to the power inside the lens area^28^. The result of this calculation is shown in Fig. S1 in the supplementary section.

**Figure 3:**
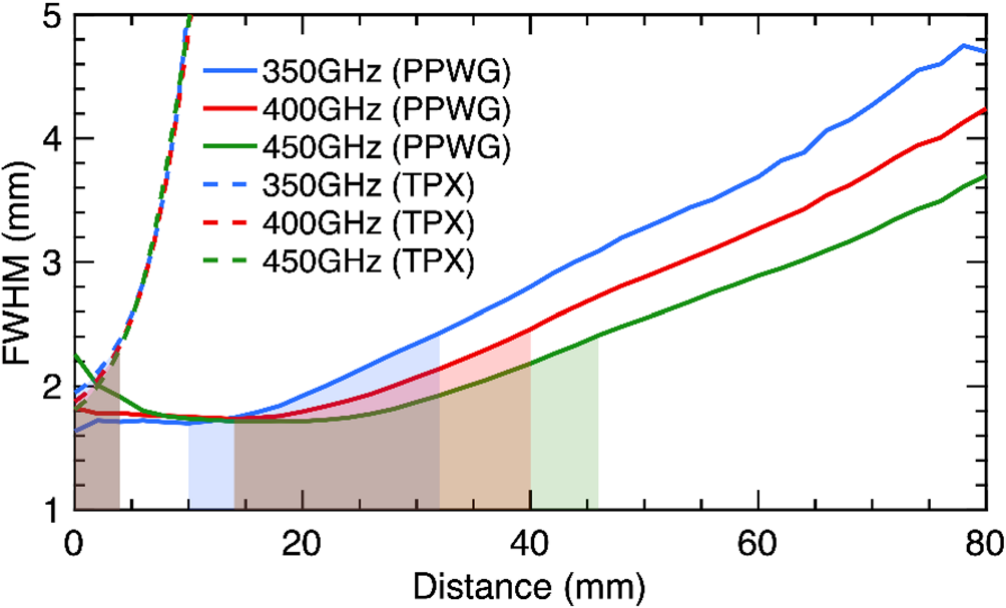
Experimental Full-Width-Half-Maximum (FWHM) as a function of distance and frequency for the TPX lens and the metallic axicon. The shaded regions denote the depth of focus of the two optical elements. These shaded regions star at the minimum of the FWHM curve (${FWHM}_{min}$) and end at the point where the FWHM reaches a value of $\sqrt{2}*{FWHM}_{min}$. From these curves the improved capabilities of the PPWG-based axicon are demonstrated.

To quantify the DOF as a function of frequency and distance, a Gaussian curve was fitted along the vertical axis in Fig. 2 for every z position. From the fitting the Full-Width-Half-Maximum (FWHM) is retrieved and plotted. These results are presented in Fig. 3.

Additionally, the DOF of a conventional TPX lens is presented for comparison. The TPX lens has a diameter of 38.1 mm and focal length of 50 mm. The shaded regions in Fig. 3 represent the DOF region. The shaded regions start from the minimum point of the FWHM curve (${FWHM}_{min}$) for each frequency and continue to the point on the curve which reaches $\sqrt{2}$ times this minimum value ($\sqrt{2}$*${FWHM}_{min}$). The results clearly demonstrate the improved capabilities of the proposed technology compared to conventional TPX optics as the DOF of the metallic axicon is 16 times higher than that of the TPX lens. Furthermore, because Eq. 1 states that the refractive index of the lens depends on the separation between plates, the proposed approach can be tuned for the frequency range of interest by simply changing this distance, an enormous advantage compared to conventional optics.

**Figure 4:**
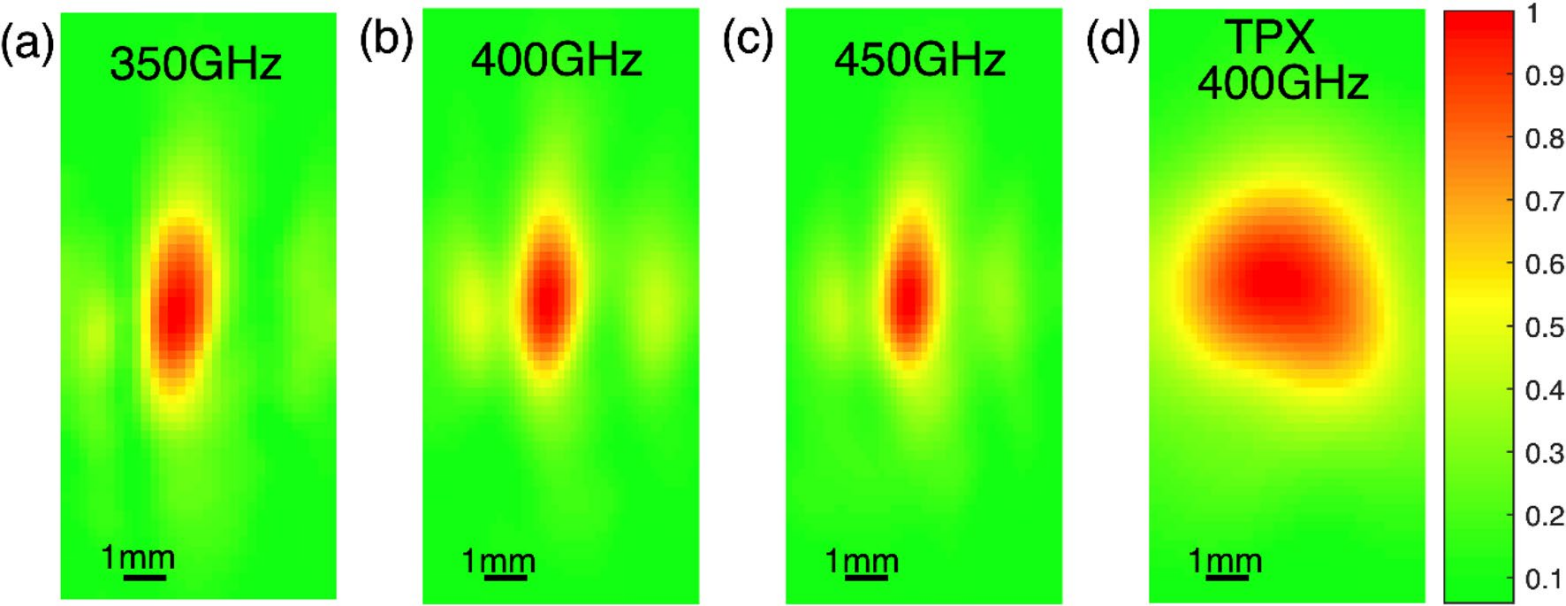
Electric field cross section at the focal point of the metallic axicon at (**a**) 350 GHz, (**b**) 400 GHz and (**c**) 450 GHz. (**d**) cross section of the TPX lens at 400 GHz. The false color images represent the amplitude of the E-field at a particular frequency.

In addition to the longitudinal measurements, in Fig. 4 transverse images taken at the focal distance are presented. These measurements were taken by mounting the detection stage in Fig. 1 on a pair of translational stages and raster scanning along the x–y plane at the focal point of each lens. In Fig. 4 three different frequencies are shown, 350 GHz, 400 GHz and 450 GHz. For comparison, the focal spot of the TPX lens at 400 GHz is also shown. Due to the fact that the variation of the focal spot of the TPX lens at these particular three frequencies is negligible, as demonstrated in Fig. 3, we only show the result at 400 GHz.

From the figure above it is clear that the PPWG based axicon achieves a smaller focal spot compared to the TPX optics, reducing the focal spot size by almost 25%. The elongated beam shown is a consequence of the 1-D geometry of the metallic axicon. Additionally, subtle side lobes are visible in Fig. 4a–c which are the result of the triangular shape of the axicon, generating a 1D Bessel beam along the *y*-direction. This is demonstrated in the supplementary section.

**Figure 5:**
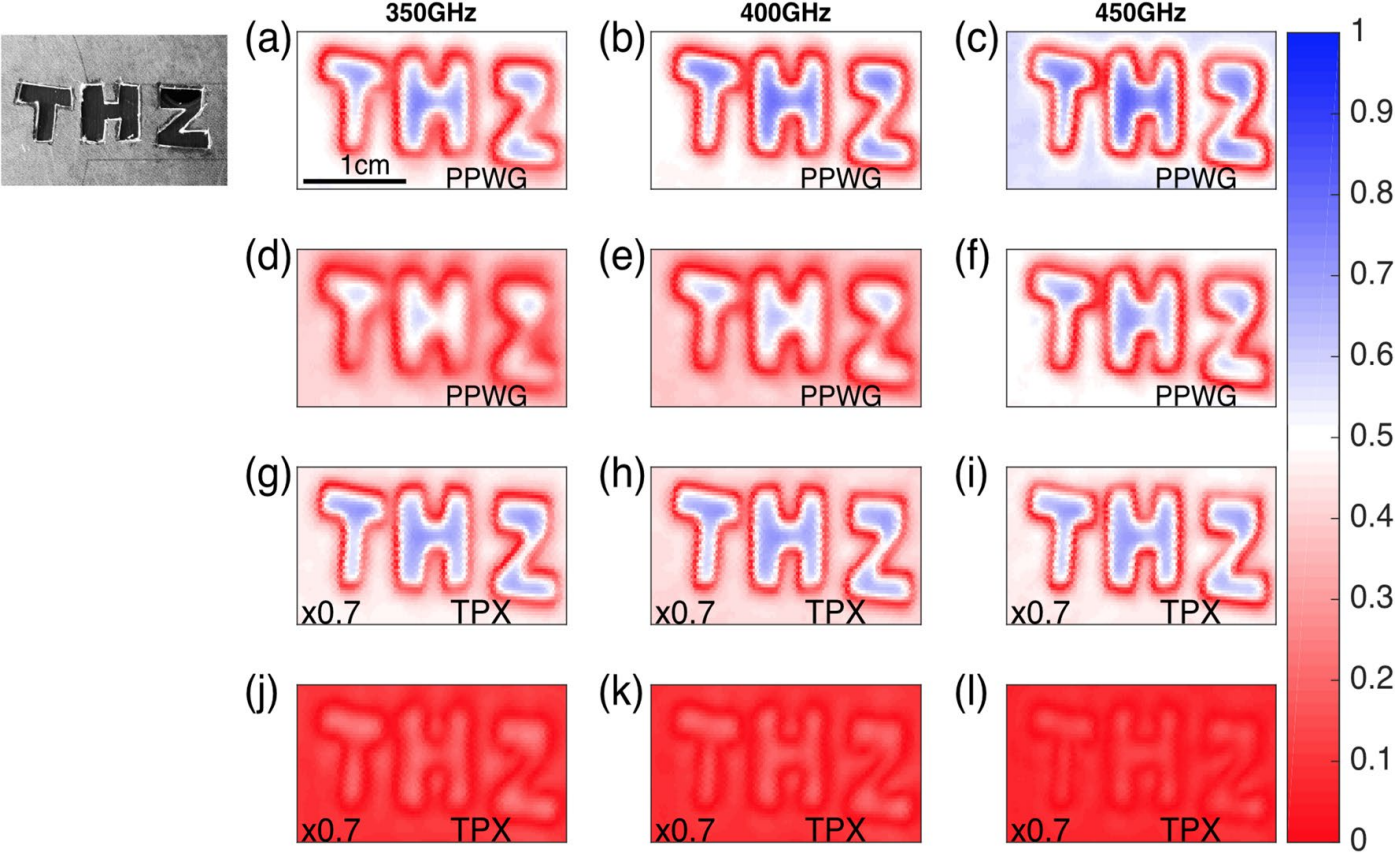
Image reconstruction at the focal point of the PPWG axicon at (**a**) 350 GHz, (**b**) 400 GHz and (**c**) 450 GHz. (**d**–**f**) Image reconstruction 3 cm after the focal point of the PPWG axicon at the same frequencies. In (**g**–**i**) the reconstruction was obtained using the TPX lens and placing the sample at its focal position. Finally, in (**j**–**l**) the sample was translated 1 cm beyond the focal point of the TPX lens. Inset, photograph of the sample.

In order to prove the performance of the proposed technology for a practical application, a transverse image of a transparent sample was taken in transmission using the same setup shown in Fig. 1. The sample consists of the message “THz” cut out from a 1 mm thick cardboard card. This is shown in Fig. 5 on the left of panel (a). The THz image was taken by mounting the sample on a couple of translational stages moving along the x–y directions while the emitter and detections stage were kept fixed in transmission as shown in Fig. 1c. The sample was placed at the focal position of the PPWG axicon and the acquisition stage was moved in a transverse plane along the x–y axis. The results for the reconstruction of the sample at 350 GHz, 400 GHz and 450 GHz are shown in Fig. 5a–c, respectively. Afterwards, the sample was moved 30 mm beyond the focal point of the PPWG axicon and the same procedure was repeated. These results are presented in Fig. 5d–f for the same frequencies. In order to compare the performance of the proposed PPWG technology, the metallic axicon was removed and the sample was measured using the TPX lenses. Figure 5g–i present the reconstruction of the sample at the focal position of the plastic lens. Subsequently, the sample was moved 10 mm beyond the focal position and we raster scanned the sample again. The results are presented in Fig. 5j–l.

By comparing the panels of Fig. 5, the improved capabilities of the PPWG axicon are clear. The sample was reconstructed within a range of 3 cm beyond the focal position of the metallic axicon, while the TPX lens produces a very low contrast image even 1 cm away from its focal spot. These results were predicted from Fig. 3 in which a rapid increase in the FWHM of the TPX lens was found within a short distance (4 mm) while for the PPWG a slower increase of the FWHM was demonstrated. Additionally, Fig. 3 also successfully explains the blurred image of the sample at 350 GHz using the PPWG axicon; the DOF at 350 GHz is less than 30 mm, reducing the quality of the reconstruction of the image at that frequency. The panels of the results of the TPX scan were multiplied by a factor of 0.7 in order to match the color bar for the PPWG axicon results. The results presented in Fig. 5 unambiguously demonstrate the superior capabilities of the PPWG technology over conventional plastic lenses.

In conclusion, in this work we present a 1D metallic axicon based on PPWG technology. This axicon presents improved capabilities having a DOF ten times larger than the TPX lenses commonly used in THz systems. Additionally, due to the triangular surface, the axicon is capable of focusing the radiation in a smaller region than the TPX lens. Finally, the use of the proposed technology clearly demonstrates than, within a range of 3 cm, the axicon preserves its resolution capabilities for a range of frequencies between 350 to 450 GHz, creating potential uses for tomographic 3D reconstruction applications. The axicon was fabricated using stainless steel sheets, making it robust, easy to fabricate and additionally low cost. The operation of the proposed axicon at different frequencies is easily possible by changing the spacing between plates. Additionally, changing the space between plates makes it possible to increase/decrease the DOF and increase/decrease the spot size of the beam, adjusting the performance to the preferences of the operator. PPWG technology has proved useful in the manufacture of novel THz devices and we predict wide implementation of the proposed technology in the next generation of THz networks and THz imaging systems.

## Supplementary Information

Supplementary Information

## Data Availability

The data presented in this paper is available at: https://figshare.com/articles/dataset/Metallic_lens_paper/13066031.
