# Supplementary material for: Low cost and long-focal-depth metallic axicon for terahertz frequencies based on parallel-plate-waveguides
Source: Sci Rep. 2021 Feb 4;11:3005. doi: 10.1038/s41598-021-82503-x (PMC7862276; doi:10.1038/s41598-021-82503-x)
Supplement: Supplementary file 1 — Supplementary Information [file 41598_2021_82503_MOESM1_ESM.pdf]

Low cost and long-focal-depth metallic axicon for terahertz frequencies based on parallel-plate-waveguides

A.I. Hernandez-Serrano, and Emma Pickwell-MacPherson

Supplementary material

Table S1. Summary of the different diffractive optics elements discussed in the paper.

Reference	Optical element	Tunable	Fabrication	Bandwidth	Depth of focus
12-13	PPWG lens	Yes	Machining<br>Chemical etching	Single-frequency	Below 10 mm
19	Kinoforms	No	3D printing<br>Machining	Within 300GHz	Below 10 mm
21-22	Metalens	No	Photolitography	Within 200GHz	Below 10 mm
14-18	Fresnel zone plate	No	3D printing<br>Chemical etching<br>Photolithography	Below 100GHz	Below 15 mm
20	Diffractive axicon	No	3D printing<br>Machining	Single-frequency	230λ<br>(230mm at 300GHz)
23-25	Refractive axicon	No	3D printing<br>Machining	Single-frequency	Below 270mm

1 Focussing efficiency calculation

Focussing efficiency calculated by the ratio of power inside a 3xFWHM region to the power inside the lens area [1]:

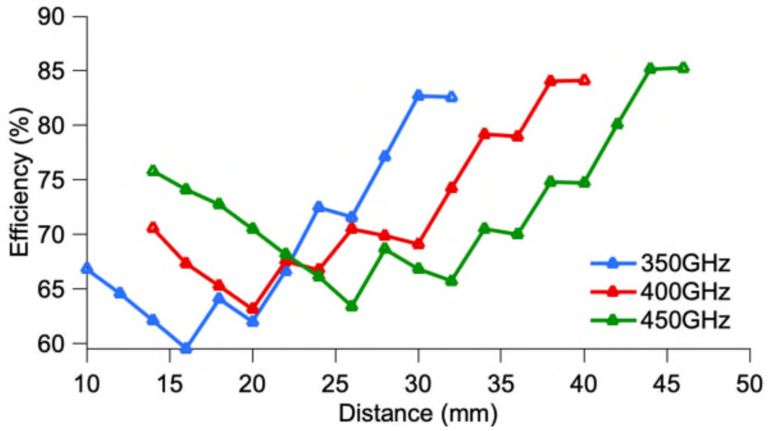

Figure S1. Focusing efficiency calculation along the depth of field (DOF).

2 Bessel beam formation

According with the geometry shown in Fig.1, the phase acquired by a beam passing through the metallic axicon relative to free space is given by:

$$\varphi = k_0(n-1)y tan(\theta/2)$$

where y is the vertical coordinate, k_0 is the free space propagating vector, n the effective refractive index of the PPWG structure and θ the apex angle of the axicon. Inserting this into the Fresnel-Kirchhoff diffraction formula [2]

$$E(x_i, y_i) \propto \iint_{-\infty}^{\infty} E_0 e^{-ik_0(n-1)y_0 tan(\theta/2)} e^{-i2\pi(f_x x_0+f_y y_0)} dx_0 dy_0$$

$$E(x_i, y_i) \propto \iint_{-\infty}^{\infty} E_0 e^{-iay_0} e^{-i2\pi(f_x x_0+f_y y_0)} dx_0 dy_0, \qquad (1)$$

in which “i” and “o” coordinates refer to image and object plane, respectively and

$$a = k_0(n-1)tan\left(\frac{\theta}{2}\right).$$

By noticing that the axicon is symmetric along the x direction (1D axicon), eq (1) can be reduced to:

$$E(x_i, y_i) \propto \int_{-L}^{L} E_0 e^{-iay_0} e^{-i2\pi(f_y y_0)} dy_0 = \int_{-L/2}^{L/2} E_0 e^{-iay_0} e^{-i\omega_y y_0} dy_0 \qquad (2)$$

in which L is the size of the axicon along the vertical direction. Evaluation of this las integral gives:

$$E(x_i, y_i) \propto sinc(L/2(a+\omega_y)).$$

This unnormalized sinc function represents the zeroth-order Bessel function, therefore, the beam generated by the PPWG axicon is a Bessel beam.

[1] Banerji, S., Meem, M., Majumder, A., Sensale-Rodriguez, B., & Menor, R. Extreme-depth-of-focus imaging with a flat lens. Optica, 7(3), 214-217, (2020). doi: 10.1364/OPTICA.384164.

[2] Iizuka, K. (2002). Elements of Photonics, Volume 1: In Free space and Special Media (Vol.41). John Wiley & Sons.
